# Adverse childhood experiences and 8-year trajectories of depressive symptoms in community-dwelling older adults: Results from the English Longitudinal Study of Ageing

**DOI:** 10.1192/j.eurpsy.2024.690

**Published:** 2024-08-27

**Authors:** A. K. C. Chau, J. K. N. Chan, H. W. Fung, E. T. C. Lai, I. Y. Y. Ho

**Affiliations:** ^1^Institute of Health Equity, The Chinese University of Hong Kong; ^2^Department of Psychiatry, School of Clinical Medicine, LKS Faculty of Medicine, The University of Hong Kong; ^3^Department of Social Work, Hong Kong Baptist University; ^4^Department of Medicine and Therapeutics, Faculty of Medicine, The Chinese University of Hong Kong, Hong Kong, Hong Kong

## Abstract

**Introduction:**

The negative impact of adverse childhood experiences (ACEs) on mental health has been well documented. While most of the evidence comes from samples of adolescents and young adults, few studies have investigated whether ACEs contribute to poorer mental health among older adults. In particular, depressive symptoms are common in old age, and they display heterogeneous patterns of development across individuals. Therefore, it is important to examine if ACEs are predictive of distinct trajectories of depressive symptoms among older adults.

**Objectives:**

Using longitudinal data from the English Longitudinal Study of Ageing (ELSA), we aimed to examine if ACEs could differentiate between distinct trajectories of depressive symptoms over eight years in community-dwelling older adults.

**Methods:**

Participants from ELSA aged 60 or above who reported no psychiatric diagnoses and completed the items of ACEs at baseline (wave 3) were included in the current study. Nine items of ACEs were subject to a principal component analysis to identify the underlying subtypes. Data of depressive symptoms from waves 3 to 7 (2-year apart), assessed with the 8-item Centre for Epidemiological Studies Depression Scale, were extracted for modelling the distinct trajectories using latent class growth analysis. The trajectories were predicted by subtypes of ACEs using multinomial logistic regression, adjusting for childhood socioeconomic status, sex, age and ethnicity.

**Results:**

The final sample consisted of 4057 participants (54.4% female, mean age= 71.34 (SD= 8.14)). We identified five trajectories of depressive symptoms (Figure 1): ‘low stable’ (73.4%), ‘increasing then decreasing’ (9.9%), ‘high decreasing’ (7.1%), ‘high stable’ (5.7%) and ‘moderate increasing’ (4.0%). Four subtypes of ACEs (i.e., sexual abuse, separation from natural parents, family dysfunction and physical assault) were evident. Compared to the ‘low stable’ group, higher levels of family dysfunction were reported in the ‘increasing then decreasing’ (aOR = 1.35, 95% CI [1.10 - 1.66], p = .012), ‘high stable’ (aOR = 1.59, 95% CI [1.30 - 1.96], p < .001) and ‘moderate increasing’ (aOR = 1.55, 95% CI [1.18 - 2.04], p = .011) groups. The ‘high stable’ group also reported a higher level of separation from natural parents than the ‘low stable’ group (aOR = 1.34, 95% CI [1.04 - 1.72], p = .047). Sexual abuse and physical assault did not predict any group differences.

**Image:**

**Figure:**
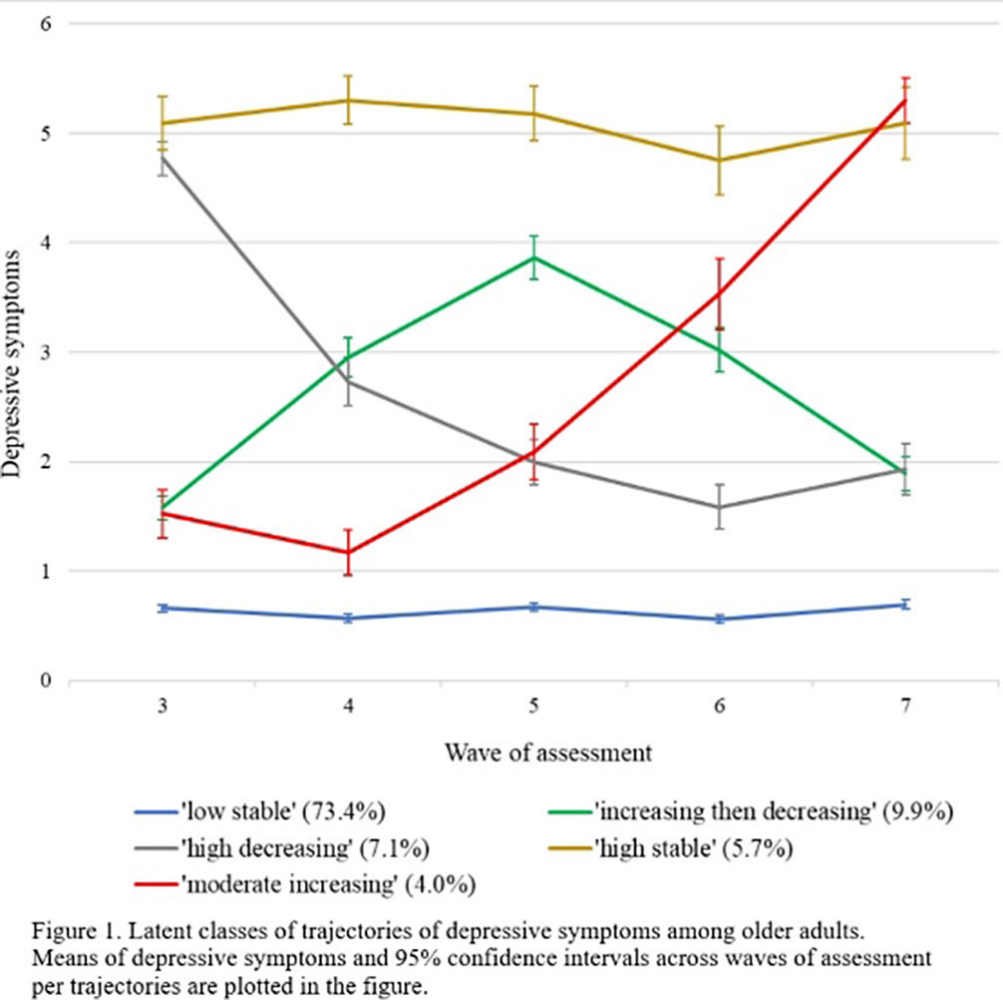

**Conclusions:**

Distinct trajectories of depressive symptoms among older adults were predicted by family dysfunction in childhood. Our findings suggested that the negative impact of ACEs on mental health may extend beyond adolescence and young adulthood into the old age.

**Disclosure of Interest:**

None Declared

